# OBESITY AND MIDLIFE PHYSICAL FUNCTIONING IN TWO BRITISH BIRTH COHORTS: THE MEDIATING ROLE OF PHYSICAL INACTIVITY

**DOI:** 10.1093/geroni/igz038.191

**Published:** 2019-11-08

**Authors:** Snehal Pinto Pereira, Bianca L De Stavola, Nina T Rogers, Rebecca Hardy, Rachel Cooper, Chris Power

**Affiliations:** 1 University College London, London, England, United Kingdom; 2 UCL GOS Institute of Child Health, London, England, United Kingdom; 3 UCL Research Department of Epidemiology & Public Health, London, England, United Kingdom; 4 MRC Unit for Lifelong Health and Ageing, London, England, United Kingdom; 5 NA, London, England, United Kingdom

## Abstract

Associations between obesity and physical inactivity are bi-directional. Both are associated with physical functioning (PF) but whether obesity influences PF via inactivity is unknown. We investigated whether mid-adult obesity trajectories were associated with subsequent PF and mediated by inactivity in the 1946 National Survey of Health and Development (1946-NSHD; N=2,427) and the 1958 National Child Development Study (1958-NCDS; N=8,674). Estimated randomised-interventional-analogue natural direct (rNDE), indirect (rNIE) and total (rTE=rNDExrNIE) effects of obesity trajectories on PF via inactivity are expressed as risk ratios. In 1946-NSHD, rTE of incident obesity at 43y (vs never) on poor PF=2.32(1.13,3.51); at 53y=1.53(0.91,2.15). rNIEs via inactivity were 1.02(0.97,1.07) and 1.02(0.99,1.04) respectively. Estimated rTE of persistent obesity from 36y=2.91(1.14,4.69), with rNIE of 1.03(0.96,1.10). Longer obesity duration was associated with increased risk of poor PF. Inactivity played a small mediating role. Findings reinforce the importance of preventing and delaying obesity onset to protect against poor PF.

